# A nomogram model integrating transcriptomics and clinical features for predicting the risk of cervical intraepithelial lesion progression

**DOI:** 10.3389/fonc.2026.1794010

**Published:** 2026-08-12

**Authors:** Shikang Qiu, Qiannan Wang, Siqi Guan, Hanxiao Ding, Limin Feng

**Affiliations:** 1 Department of Gynecology, The First Affiliated Hospital of Shandong First Medical University & Shandong Provincial Qianfoshan Hospital, Jinan, China; 2 Department of Gynecology, Beijing Tiantan Hospital, Capital Medical University, Beijing, China

**Keywords:** biomarkers, cervical intraepithelial lesion, nomogram, transcriptomics, risk prediction

## Abstract

**Objectives:**

To identify molecular and clinical predictors of cervical lesion progression and construct an integrated nomogram model for personalized risk assessment in patients with LSIL.

**Methods:**

Transcriptomic data from the GEO database were analyzed for differentially expressed genes (DEGs). GO and KEGG enrichment analyses identified biological functions and pathways, while STRING network screened candidate core genes. Immunohistochemistry was performed on patient samples to evaluate the expression of key proteins associated with progression. A prediction model using Cox regression and a random forest algorithm was developed based on clinical data and immunohistochemical markers, with model discrimination and potential clinical net benefit assessed by ROC and decision curve analysis (DCA).

**Results:**

We identified 221 upregulated and 161 downregulated DEGs. Key progression-related genes included *CDKN2A, CALML5, GINS2, KCNH1, MCM2, MKI67*, and *ESR1*. Immunohistochemical validation in 155 patients showed that Eag1, p16INK4a, and Ki-67 correlated with poor outcomes. Colposcopic lesion involvement across cervical quadrants showed an inverse association with progression, while bacterial vaginosis was a risk factor. The prediction model showed favorable discrimination (AUC: 0.914/0.753 for 1-year and 0.941/0.769 for 2-year progression). DCA suggested potential clinical net benefit across selected threshold probabilities. External validation is needed to confirm the generalizability and clinical utility of the model.

**Conclusion:**

This study identifies candidate biomarkers and clinical factors associated with cervical lesion progression and provides a preliminary prediction model for risk stratification.

## 1 Introduction

Cervical squamous cell carcinoma (SCC) arises from long-term chronic infection of cervical epithelial cells by human papillomavirus (HPV), leading to dysplastic transformation and malignant progression (1, 2). Cervical intraepithelial neoplasia (CIN) represents a premalignant stage of SCC and is classified into low-grade squamous intraepithelial lesion (LSIL) and high-grade squamous intraepithelial lesion (HSIL) based on the severity of dysplasia. HSIL carries a high risk of progression to invasive carcinoma and typically requires surgical intervention, whereas LSIL has a lower malignant potential and is often managed with regular surveillance. However, delayed cervical screening may result in occult progression of LSIL, significantly increasing the risk of invasive carcinoma (3). The risk of LSIL progression varies among individuals, with some cases regressing spontaneously while others progress to HSIL or even invasive carcinoma (4). The underlying mechanisms governing this process remain largely unknown. Therefore, investigating the molecular mechanisms involved in CIN progression and identifying potential biomarkers for risk prediction are critical for early intervention and reducing the incidence of invasive cervical cancer.

In recent years, transcriptomic technologies, including mRNA sequencing and microarray analysis, have been widely applied in cancer research, facilitating the identification of key genes and signaling pathways associated with disease progression (5, 6). In the context of cervical diseases, these technologies have been utilized to identify differentially expressed genes (DEGs) and regulatory networks implicated in CIN progression. A transcriptomic study involving HSIL and invasive cervical carcinoma samples revealed significant cellular heterogeneity and differential expression of key molecules, such as the tumor suppressor *SLC5A8* and the cell adhesion molecules *CDH16* and *CDH17*, in the context of high-risk HPV (HR-HPV) infection. Additionally, the study highlighted immune cell heterogeneity within the tumor stroma and variations in the immune microenvironment across different CIN grades (7). These findings provide new insights into the application of bioinformatics-based transcriptomic analysis for constructing CIN progression risk prediction models.

Beyond genetic factors, CIN progression is influenced by multiple external factors. Carcinogenesis is a complex process involving multiple factors, pathways, and steps, driven by the interaction of environmental and genetic determinants (8, 9). Consequently, individual clinical characteristics may play a crucial role in disease development and prognosis. Previous studies have suggested that factors such as patient age, smoking history, HPV genotype, viral load, and immune status may be associated with CIN progression (10–12). However, the predictive value of these factors remains limited due to small study cohorts and the heterogeneity of variable selection. Therefore, further research is needed to incorporate both clinical and genetic information to optimize model construction and improve the accuracy and applicability of CIN progression prediction.

With advancements in clinical diagnostics and transcriptomic technologies, a wealth of publicly available clinical and molecular data on CIN patients has become accessible, providing a foundation for investigating CIN progression-related factors. In this study, we focused on transcriptomic alterations during CIN progression. We performed differential expression analysis on transcriptomic datasets comprising LSIL and HSIL samples and extracted mRNA expression datasets with CIN patient prognostic information for validation. Following the identification of key molecular biomarkers associated with disease progression, we conducted immunohistochemical validation using pathological specimens from CIN patients at our center, collected relevant clinical data, and tracked disease progression outcomes. By integrating key molecular markers with clinical characteristics, we developed a risk prediction model for CIN progression, which may contribute to elucidating the evolutionary trajectory of CIN and provide a theoretical basis for early cervical cancer diagnosis and personalized treatment strategies.

## 2 Materials and methods

### 2.1 Public data acquisition

CIN-related gene expression datasets were retrieved from the GEO database (NCBI) using relevant search terms, restricted to human studies with array or high-throughput sequencing data. Datasets analyzing differential gene expression required both LSIL and HSIL samples with HR-HPV infection, while those for CIN progression validation included samples below HSIL with documented progression outcomes. Three Affymetrix U133 Plus 2.0 datasets (GSE27678 (13–15), GSE63514 (16), GSE75132 (17)) were selected.

### 2.2 Public data processing and molecular marker selection

Differential expression analysis was performed using GEO2R based on the submitter-supplied processed Series Matrix data. For microarray datasets, GEO2R applies the GEOquery and limma packages with empirical Bayes moderation. GO and KEGG analyses identified enriched functions and pathways (p < 0.05).

A PPI network was built using STRING (confidence score ≥ 0.7) and analyzed for key nodes based on degree, betweenness, and closeness centrality (18, 19). These centrality measures were used as descriptive topological indices for candidate hub-gene prioritization. Candidate hub genes identified from the PPI analysis were further evaluated in the GSE75132 dataset, which included documented CIN progression outcomes. Their associations with CIN progression were assessed using univariate and multivariate Cox regression, with Schoenfeld residuals used to assess model assumptions. Kaplan-Meier curves and ROC analysis were performed to evaluate progression-related predictive performance. Genes associated with CIN progression and showing an AUC > 0.7 were considered candidate progression-related molecular markers. Based on Cox regression results, AUC performance, biological relevance, published evidence, and feasibility of immunohistochemical assessment in clinical tissue samples, final candidate molecular markers were selected for immunohistochemical validation. The immunohistochemical staining results of the selected markers were then incorporated as molecular variables in the clinical prediction model.

### 2.3 Clinical model development

A real-world cohort study was conducted to develop a prediction model for LSIL progression in HR-HPV-infected patients. Eligible patients were prospectively enrolled, with clinical data collection, pathological sample preservation, and a two-year follow-up. Candidate clinical predictors were selected based on clinical relevance and prior knowledge of CIN progression, including HPV infection-related factors, vaginal infection status, colposcopic findings, and routinely collected demographic and clinical characteristics. These clinical predictors were subsequently analyzed together with IHC-derived molecular markers during feature selection and model construction.

### 2.4 Patient recruitment

Patients were recruited from the Gynecology Outpatient Clinic of Beijing Tiantan Hospital beginning in November 2021. The inclusion criteria were defined as follows: (1) Female patients, age ≥18 years; (2) PCR-confirmed HR-HPV infection (e.g., HPV 16, 18, 31, 33, or 45) with colposcopy and biopsy-confirmed LSIL; (3) written informed consent for study participation and follow-up. The exclusion criteria were defined as follows: (1) prior cervical cancer or related treatments; (2) pregnancy or lactation; (3) other malignancies or severe systemic diseases (e.g., HIV infection, active hepatitis); (4) inability to complete follow-up. Cases with missing values in variables required for a given analysis were excluded from the corresponding analysis.

### 2.5 Clinical data collection and sample processing

The collected clinical information included the following: (1) Demographic characteristics: age, BMI, marital status, and parity; (2) Medical history: menstrual status, high-risk HPV genotypes, ThinPrep cytology test (TCT) results, vaginal infection history (bacterial vaginosis, vulvovaginal candidiasis), prior cervical lesions, and family history of cancer; (3) Colposcopic findings: transformation zone type, extent of lesions, and presence of vaginal involvement. The extent of cervical lesions was recorded according to the number of involved cervical quadrants.

Pathological samples were obtained via colposcopy-guided biopsy using disposable cervical biopsy forceps, immediately fixed in 10% neutral formalin, paraffin-embedded after 24 hours. Formalin-fixed paraffin-embedded tissue sections were prepared for subsequent immunohistochemical analysis.

### 2.6 Patient follow-up and disease progression assessment

Patients were followed for two years, with clinical reassessment every six months, including gynecological examination, TCT, and HPV genotyping. Colposcopy and biopsy were performed when clinically indicated.

Disease progression was defined as histologically confirmed CIN grade elevation (e.g., CIN1 to CIN2/3 or invasive carcinoma). Cases with stable or regressive lesions were classified as non-progression. All clinical and laboratory data were recorded in a structured electronic database and verified by two independent reviewers.

### 2.7 Immunohistochemistry

Cervical biopsy samples from 155 patients were stained for p16 (1:100, 725-4713, Roche), Ki-67 (1:200, M7240, Agilent), and Eag1 (1:500, NBP1-84935, Novus). Staining intensity was scored on a 4-point scale (0 = negative to 3 = strong). Images were captured using an optical microscope and analyzed with NIS-Elements software. For each patient, three representative high-power fields (100 epithelial cells each) were evaluated. Histological assessments were independently performed by two experienced pathologists blinded to patient outcomes.

### 2.8 Statistical analysis

Patients were randomly assigned to training and validation cohorts at a 4:1 ratio. Continuous variables were presented as medians (interquartile range). Data distribution was assessed for normality using the Shapiro-Wilk test. Depending on distribution, comparisons were made using either the Student’s t-test or the Mann-Whitney U test. Categorical variables were analyzed using the chi-square test or Fisher’s exact test. Feature selection was performed using a random forest classifier with the Boruta algorithm. Candidate molecular variables derived from IHC assessment and candidate clinical variables selected based on clinical relevance were jointly evaluated using Boruta-based feature selection. Variables identified as important by Boruta were considered for subsequent Cox regression model construction. Kaplan-Meier survival analysis was conducted to estimate cumulative risk over time. Key predictors were incorporated into a Cox regression model, with a nomogram constructed for risk estimation. After final model construction, the number of progression events, the number of predictors in the final model, and the approximate events-per-variable ratio were calculated to assess model complexity relative to the available outcome events. Model performance was evaluated using ROC curves, and AUC values were used to quantify discriminatory ability. Decision curve analysis (DCA) was conducted to assess potential clinical net benefit. Given the relatively small validation cohort, additional internal validation was performed using Efron’s leave-one-out bootstrap with 100 resamples. Model discrimination was further assessed using the leave-pair-out bootstrap approach. Statistical significance was set at p < 0.05, and all analyses were performed using R (version 4.2.2).

## 3 Results

### 3.1 Sample selection and differential expression analysis

**Figure 1:**
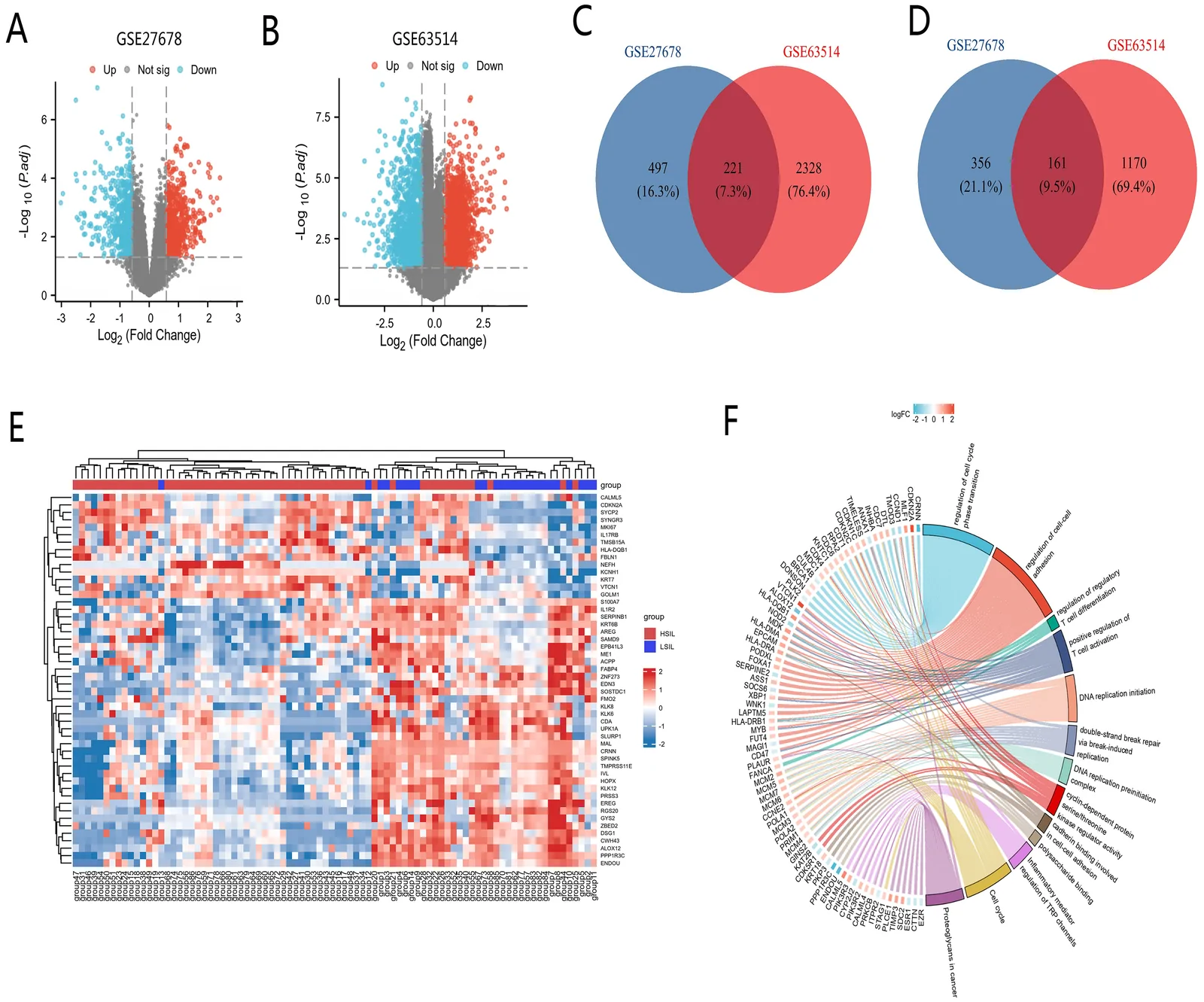
Preliminary screening and analysis of DEGs. **(A)** Volcano plot of DEGs in the GSE27678 dataset. **(B)** Volcano plot of DEGs in the GSE63514 dataset. **(C)** Venn diagram of upregulated DEGs. **(D)** Venn diagram of downregulated DEGs. **(E)** Heatmap of the top 50 DEGs from the intersection of differential analysis results in the datasets. **(F)** Enrichment results of DEGs from two patient groups in the dataset and chord diagram of the expression of corresponding core genes.

A subset of samples from two datasets was used for DEG analysis: GSE27678 (11 LSIL, 21 HSIL) and GSE63514 (14 LSIL, 40 HSIL). Volcano plots illustrate the results, with 874 upregulated and 658 downregulated genes in GSE27678 (**Figure 1A**) and 3,201 upregulated and 1,780 downregulated genes in GSE63514 (**Figure 1B**).

Intersection analysis identified 221 shared upregulated DEGs (**Figure 1C**) and 161 shared downregulated DEGs (**Figure 1D**). The top 50 intersecting DEGs, visualized in a heatmap (**Figure 1E**), showed distinct expression patterns between HSIL and LSIL, including *CDKN2A* (related to cell cycle regulation), *HLA-DQB1* (related to tumor immune evasion), and *KRT8* (related to EMT).

GO and KEGG enrichment analyses (**Figure 1F**) suggested enrichment of biological processes and pathways potentially associated with in LSIL-to-HSIL progression. GO terms include cell cycle phase transition, cell-cell adhesion, and regulatory T cell differentiation. KEGG analysis revealed enrichment in inflammatory mediator regulation of TRP channels and proteoglycans in cancer, with core genes predominantly upregulated, suggesting their potential role in disease progression.

### 3.2 Construction of PPI network based on DEGs from different cervical lesions and focus on core genes

**Figure 2:**
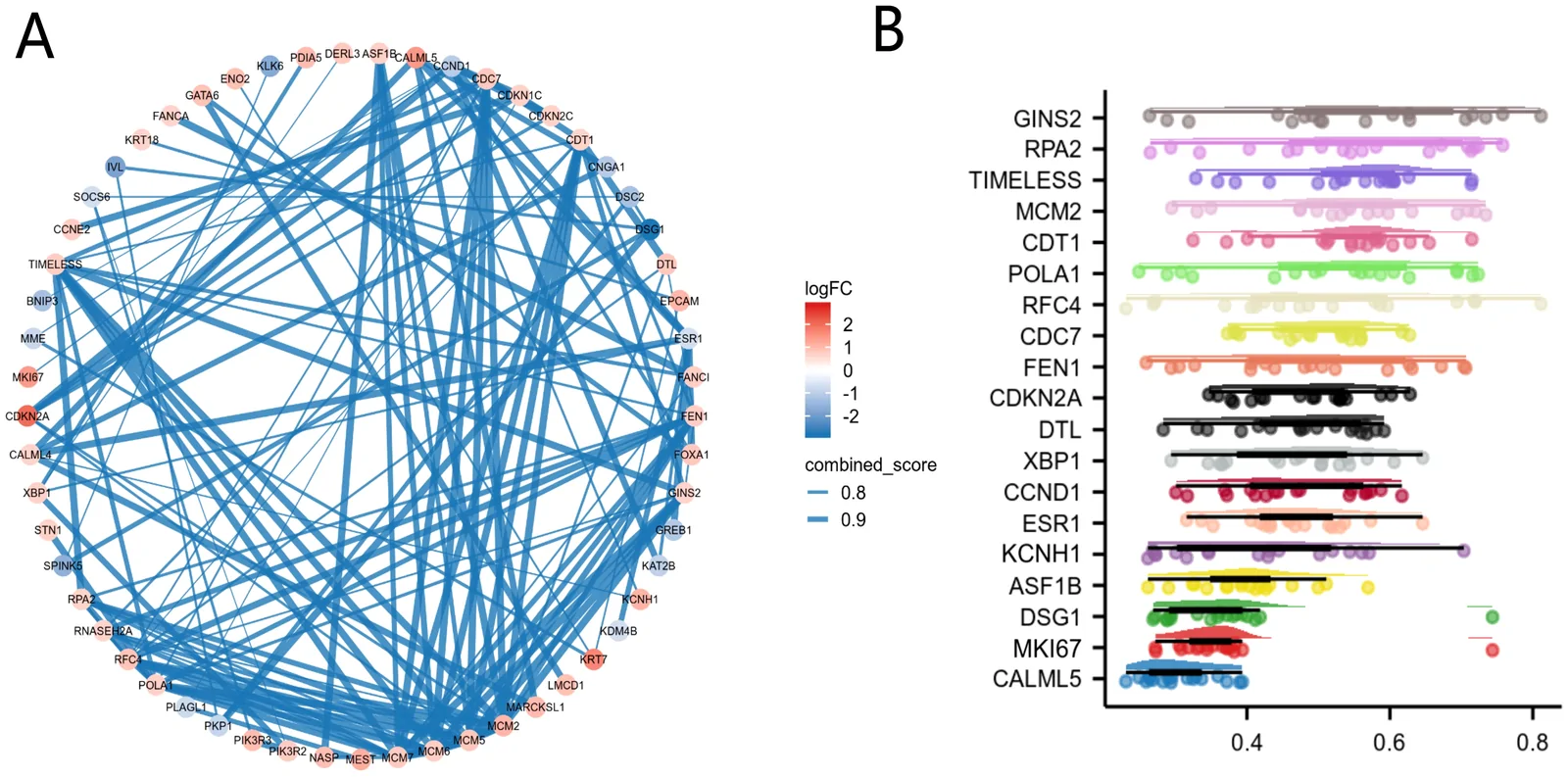
Interactions of DEGs. **(A)** Interaction network of molecules with a confidence score of 0.7. **(B)** Importance ranking of high-confidence node molecules based on friends analysis.

To further identify candidate hub genes, a protein-protein interaction (PPI) network was constructed based on DEGs using the STRING database. **Figure 2A** shows the interactions between molecules with a confidence score > 0.7. **Figure 2B** presents the ranking of high-confidence nodes based on network centrality analysis, with higher-ranked genes occupying more central positions in the PPI network.

A total of 19 core genes with high topological importance were identified. Among these, *GINS2, RPA2, TIMELESS, MCM2, CDT1, POLA1, RFC4, CDC7, FEN1, CDKN2A, DTL, XBP1, KCNH1, ASF1B, MKI67*, and *CALML5* were upregulated, while *DSG1, ESR1*, and *CCND1* were downregulated. These genes have been reported to be involved in biological processes relevant to lesion progression and tumorigenesis, including cell cycle regulation, cell proliferation and apoptosis, and cell-cell adhesion.

### 3.3 Core genes’ impact on cervical lesion progression using public datasets

**Figure 3:**
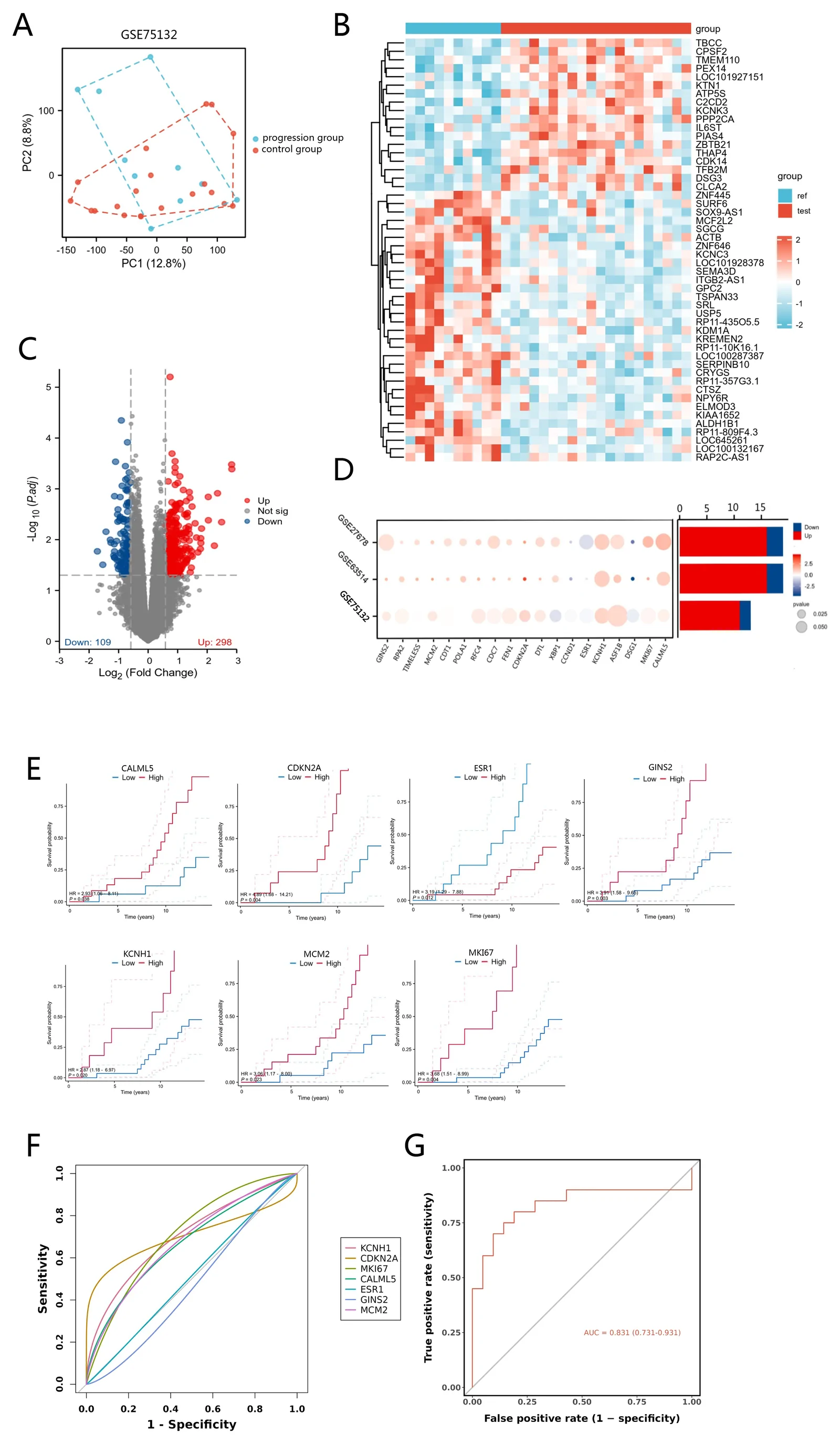
Screening and validation of core genes. **(A)** PCA plot of samples from the GSE75132 dataset. **(B)** Heatmap of the top 50 DEGs identified in GSE75132. **(C)** Volcano plot of DEGs between the progression and control groups in GSE75132. **(D)** Bubble plot of core-gene differential expression across GSE27678, GSE63514, and GSE75132, accompanied by stacked bars summarizing the numbers of upregulated and downregulated genes. **(E)** Cumulative progression-risk curves for CALML5, CDKN2A, ESR1, GINS2, KCNH1, MCM2, and MKI67, comparing low and high expression groups. **(F)** ROC curves evaluating the ability of individual genes to discriminate CIN progression outcomes. **(G)** ROC curve of the model (AUC = 0.831; 95% CI, 0.731 -0.931).

To explore the potential of core genes as disease progression predictors, we analyzed the GSE75132 dataset, which includes CIN progression outcomes. Among 30 HR-HPV-infected patients, 20 showed progression to higher-grade lesions, and 10 did not progress or regressed. The progression group showed partial separation from the control group in the PCA (**Figure 3A**). The heatmap (**Figure 3B**) highlights core genes like CDKN2A, MCM2, and ESR1, which displayed differential expression patterns between HSIL and LSIL samples. Differential expression analysis identified 298 upregulated genes (red) and 109 downregulated genes (blue) (**Figure 3C**).

**Figure 3D** shows scatter plots of core genes’ expression across three datasets, with genes like *XBP1, POLA1*, and *TIMELESS* showing inconsistencies, while others like *GINS2, RPA2, MCM2, CDKN2A, KCNH1*, and *MKI67* showed relatively consistent expression trends across all datasets. The bar chart indicates the proportion of upregulated or downregulated genes.

To assess the genes’ association with progression, Cox regression models were built for high- and low-expression groups based on median expression. **Figure 3E** shows a significant association between *CALML5, CDKN2A, ESR1, GINS2, KCNH1, MCM2*, and *MKI67* expression and progression (p < 0.05). ROC analysis (**Figure 3F**) revealed *CDKN2A, KCNH1*, and *MKI67* had AUC values >0.7, indicating potential predictive value.

Overall, the marker-selection pipeline proceeded from 382 intersecting DEGs to 19 PPI-prioritized core genes, then to 7 progression-associated genes in Cox regression analysis, and finally to 3 markers (*CDKN2A, KCNH1*, and *MKI67*) with AUC values > 0.7, which were selected for IHC validation. A multivariate model incorporating these 3 markers showed favorable discrimination (AUC = 0.831, 95% CI: 0.731-0.931) for predicting disease progression (**Figure 3G**).

### 3.4 Construction of a CIN progression prediction model using immunohistochemical scores and clinical data

To create a clinically relevant exploratory multifactorial model for predicting CIN progression, this study integrated molecular markers and clinical features using a Cox regression model. A total of 155 LSIL patients were included, with 49 progressing to higher-grade lesions and 106 showing persistent or regressing lower-grade lesions over 2 years.

**Figure 4:**
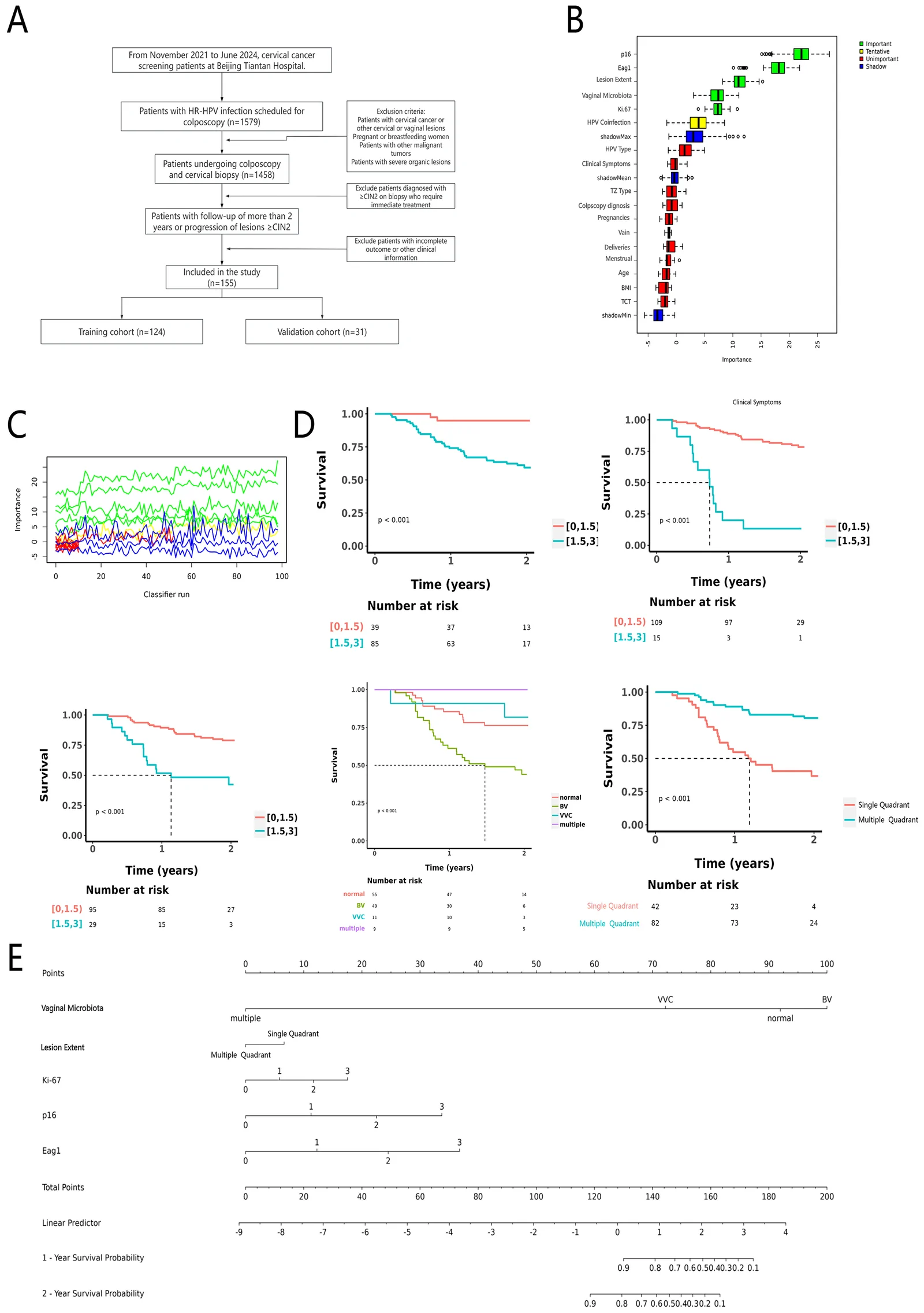
Construction of the prediction model. **(A)** Flowchart of patient screening, inclusion and exclusion, and division into training and validation cohorts. **(B)** Boruta feature-importance plot of the candidate predictors. **(C)** History plot of feature-importance scores across Boruta classifier runs. **(D)** Kaplan–Meier curves stratified by the model-derived risk score and selected clinical predictors, with corresponding numbers at risk. **(E)** Nomogram integrating vaginal microbiota, lesion extent, Ki-67, p16, and Eag1 to estimate 1- and 2-year survival probabilities.

Patients were divided into training and validation cohorts (4:1 ratio) for internal validation, as shown in **Figure 4A**. Baseline characteristics between the groups showed no significant differences (**Table 1**).

**Table 1:** Demographic and baseline characteristics of study participants.

Factor	Cohort		p-value^2^
	Training cohort, N = 124^1^	Validation cohort, N = 31^1^	
Eag1			0.837
Mean ± SD	1.84 ± 0.82	1.87 ± 0.76	
p16			0.456
Mean ± SD	0.88 ± 0.97	0.74 ± 0.89	
Ki-67			0.195
Mean ± SD	1.12 ± 0.50	1.00 ± 0.45	
Age(year)			0.290
Mean ± SD	43 ± 11	40 ± 9	
BMI			0.786
[16.7,24.9)	104 (83.9%)	27 (87.1%)	
[24.9,33.1]	20 (16.1%)	4 (12.9%)	
Clinical symptoms			0.688
None	100 (80.6%)	24 (77.4%)	
Symptomatic	24 (19.4%)	7 (22.6%)	
TCT results			0.770
NILM	46 (37.1%)	14 (45.2%)	
ASC_US	38 (30.6%)	11 (35.5%)	
LSIL	32 (25.8%)	5 (16.1%)	
ASC_H	5 (4.0%)	1 (3.2%)	
HSIL	3 (2.4%)	0 (0.0%)	
HPV type			0.742
HR-HPV (others)	76 (61.3%)	18 (58.1%)	
HPV16/18	48 (38.7%)	13 (41.9%)	
HPV coinfection			0.543
Single	87 (70.2%)	20 (64.5%)	
Multiple	37 (29.8%)	11 (35.5%)	
Menstrual status			0.312
Normal	98 (79.0%)	28 (90.3%)	
Irregular	18 (14.5%)	3 (9.7%)	
Menopause	8 (6.5%)	0 (0.0%)	
Pregnancies (n)			0.376
< 2	86 (69.4%)	24 (77.4%)	
≥ 2	38 (30.6%)	7 (22.6%)	
Deliveries (n)			>0.999
< 1	28 (22.6%)	7 (22.6%)	
≥ 1	96 (77.4%)	24 (77.4%)	
TZ type			0.255
Type 1	25 (20.2%)	5 (16.1%)	
Type 2	20 (16.1%)	9 (29.0%)	
Type 3	79 (63.7%)	17 (54.8%)	
Lesion extent			0.865
Single quadrant	42 (33.9%)	10 (32.3%)	
Multiple quadrants	82 (66.1%)	21 (67.7%)	
Vain			>0.999
None	119 (96.0%)	30 (96.8%)	
exist	5 (4.0%)	1 (3.2%)	
Vaginal microbiota			0.288
normal	55 (44.4%)	14 (45.2%)	
BV	49 (39.5%)	8 (25.8%)	
VVC	11 (8.9%)	5 (16.1%)	
multiple	9 (7.3%)	4 (12.9%)	

^1^ n (%).

^2^ Welch Two Sample t-test; Pearson’s Chi-squared test; Fisher’s exact test.

Using the Boruta algorithm, we identified five potential predictors: immunohistochemical scores for p16INK4a, Eag1, Ki-67, lesion involvement in cervical quadrants, and vaginal microbial infections. **Figures 4B, C** illustrate the importance scores and decision history of these predictors. Based on the 49 observed progression events and five predictors retained in the final Cox model, the approximate events-per-variable ratio was 9.8, which was used to describe model complexity relative to the available number of progression events. Cox regression analysis (**Figure 4D**) revealed that higher scores for p16INK4a, Eag1, and Ki-67 were associated with higher progression risk, while larger lesion areas in cervical quadrants were inversely associated with progression risk. Vaginal infections, particularly bacterial vaginosis (BV), were associated with increased progression risk, whereas other infections had no significant impact.

The final regression model (**Table 2**) indicated that BV (HR = 3.30, p = 0.003) and single-quadrant lesions (HR = 0.63, p = 0.026) were significantly associated with disease progression. p16INK4a (HR = 2.40, p = 0.025) and Eag1 (HR = 8.06, p = 0.005) immunohistochemical scores were significant predictors, while Ki-67 showed a moderate association with progression risk.

**Table 2:** Multivariate Cox regression results in the training cohort.

Factor	N	Event N	HR^1^	95% CI^1^	p-value
Vaginal microbiota					
normal	55	13	—	—	
BV	49	27	3.30	2.50, 4.25	0.003
VVC	11	2	0.26	0.05, 1.26	0.094
multiple	9	0	0.00	0.00, Inf	0.996
Lesion extent					
Single quadrant	82	16	—	—	
Multiple quadrants	42	26	0.63	0.30, 1.33	0.026
Ki-67	124	42	1.50	0.87, 2.58	0.145
p16	124	42	2.40	1.12, 3.15	0.025
Eag1	124	42	8.06	7.87, 9.76	0.005

^1^ HR, Hazard Ratio, CI, Confidence Interval.

A nomogram (**Figure 4E**) was created to visualize the model, showing the contribution of each risk factor and the predicted progression risk at 1 and 2 years.

### 3.5 Evaluation of the predictive value of the CIN progression regression model using the validation cohort

**Figure 5:**
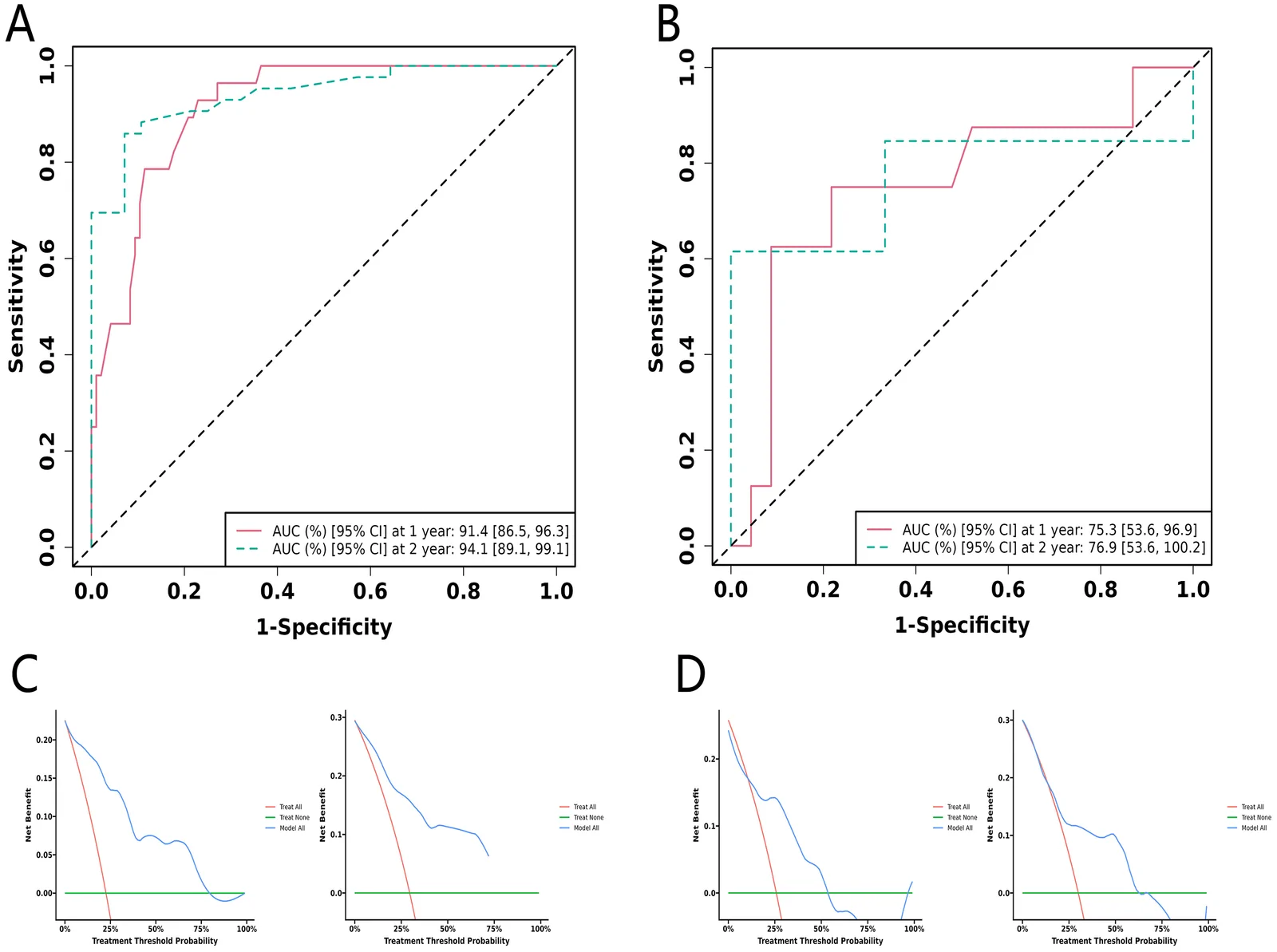
Evaluation of the prediction model. **(A)** ROC curve of the model in the training cohort; **(B)** ROC curve of the model in the validation cohort. **(C)** DCA curve of disease progression for 1 year (left) and 2 years (right) in the training cohort patients; **(D)** DCA curve of disease progression for 1 year (left) and 2 years (right) in the validation cohort patients.

To assess the predictive performance of the model, ROC curves and DCA decision curves were plotted in both the training and validation cohorts to evaluate its discriminatory ability and potential clinical net benefit. In the training cohort, the model demonstrated favorable discriminatory performance in predicting disease progression at 1 year and 2 years. **Figure 5A** shows the ROC curve areas under the curve (AUC) for disease progression at 1 year (AUC = 0.914, 95% CI: 0.865-0.963) and 2 years (AUC = 0.941, 95% CI: 0.891-0.991). **Figure 5B** shows the AUC for disease progression at 1 year (AUC = 0.753, 95% CI: 0.536-0.969) and 2 years (AUC = 0.769, 95% CI: 0.536-0.975) in the validation cohort.

DCA analysis further assessed the potential clinical net benefit of the model. **Figure 5C** shows the DCA curves for disease progression at 1 year (left) and 2 years (right) in the training cohort. **Figure 5D** shows the net benefit of the model in predicting disease progression at 1 year (left) and 2 years (right) in the validation cohort. The internally validated C-index/AUC was 0.759 (95% CI, 0.697–0.821). Given the relatively small validation cohort, these results should be interpreted as preliminary internal validation and require confirmation in larger independent cohorts.

## 4 Discussion

During CIN progression, epithelial cell atypia gradually increases, and the expression and biological functions of key intracellular proteins may be altered due to genetic mutations or changes in oncogene and tumor suppressor gene activity (20). Our differential transcriptomic analysis of lesions at different CIN grades in two independent datasets was consistent with this phenomenon. The differential expression of genes shared between both datasets may reflect molecular functional changes associated with CIN progression from low-grade to high-grade lesions, making them potential candidate predictive factors for further validation. Additionally, several DEGs identified in this study, such as *CDKN2A, HLA-DQB1*, and *KRT8*, have been reported to be involved in tumorigenesis and disease progression.

The GSE75132 dataset provides a valuable reference for preliminarily assessing the progression-related relevance of DEGs in this study. This dataset includes more than 10 years of follow-up data from women infected with HPV16, with documented disease progression outcomes and transcriptomic data from cervical swab samples of selected patients (21). Using comparative gene expression analysis and cumulative risk curve modeling, we identified seven genes significantly associated with CIN progression outcomes. These genes exhibited correlations with disease progression, suggesting that they may be related to CIN progression. The hub-gene prioritization may be influenced by the selected STRING confidence threshold and network inference strategy. Therefore, the 19 genes identified from the PPI analysis should be interpreted as candidate hub genes rather than definitive mechanistic drivers. Their stability across alternative STRING confidence thresholds or network construction methods requires further validation. Moreover, the transition from high-dimensional transcriptomic screening to clinically applicable biomarkers should be interpreted cautiously. Omics-derived biomarker discovery may be affected by dataset heterogeneity, platform-specific effects, subjective feature prioritization, and limited reproducibility (22–24). In this study, we attempted to reduce these risks by applying a stepwise selection process that integrated differential expression consistency, PPI-based prioritization, progression-related Cox analysis, ROC performance, biological plausibility, published evidence, and feasibility of IHC assessment in clinical tissue samples. Nevertheless, the selected markers should be regarded as candidate biomarkers rather than definitive progression biomarkers, and further validation in independent cohorts is required.

*CALML5*, encoding calmodulin-like protein 5, is primarily involved in epithelial cell differentiation and homeostasis (25). In certain squamous epithelial tumors, *CALML5* enhances tumor cell adhesion and proliferation, thereby increasing invasiveness (26). In this study, high *CALML5* expression was associated with an elevated risk of lesion progression, consistent with previous findings. Importantly, recent evidence indicates that *CALML5* may also serve as a clinically relevant biomarker. For instance, its aberrant expression has been linked to unfavorable prognosis in head and neck squamous cell carcinoma and cutaneous squamous cell carcinoma, suggesting potential utility in predicting disease progression and patient outcomes (27, 28). These findings support our results and raise the possibility that CALML5 may serve as a candidate biomarker for future risk-stratification studies of CIN progression, although further validation is required before clinical application.

*GINS2* and *MCM2* are key components of the DNA replication initiation complex (29). Studies have shown that their overexpression promotes the G1/S phase transition, accelerating entry into the S phase and enhancing cell proliferation. Their upregulation is frequently observed in highly proliferative tumors such as breast and ovarian cancer, making them candidates for early screening biomarkers or adjunctive markers in risk stratification (30, 31). Beyond these cancers, *GINS2* overexpression has been correlated with adverse clinical outcomes and resistance to therapy in lung adenocarcinoma and hepatocellular carcinoma (30, 32). This evidence highlights its potential value as a prognostic biomarker in cancer-related studies. Our findings that *GINS2* is upregulated in progressive CIN lesions therefore suggest that GINS2 may have potential value as a candidate marker for identifying lesions with higher progression risk, but its role in guiding surveillance or intervention strategies requires further prospective validation.

*KCNH1*, encoding Eag1, plays a role in membrane potential regulation and is involved in cell cycle control, proliferation, and migration (33). Overexpression of *KCNH1* has been reported to promote epithelial-mesenchymal transition (EMT), thereby enhancing tumor cell invasiveness (34), which may provide biological context for the association observed in our study. *MKI67*, encoding Ki-67, is a key cell cycle regulator widely used to assess proliferation activity in tumor diagnostics and prognostic evaluations (35). Its expression is strongly associated with abnormal cervical epithelial proliferation and is frequently used to differentiate LSIL from HSIL (36). Our study is among the studies suggesting its potential predictive value for CIN progression within LSIL cases. Nevertheless, the relatively large hazard ratio estimate observed for Eag1 should be interpreted with caution, as it may partly reflect statistical instability associated with the limited sample size and number of progression events.

*ESR1* was the only gene identified as being inversely associated with CIN progression risk in our study. *ESR1*, encoding estrogen receptor α, has been shown to suppress *EGFR* and *HER2* tyrosine kinase activity in estrogen-dependent tumors, altering extracellular matrix effectors and plasminogen activity to reduce EMT capacity (37). This evidence is consistent with our observation and suggests the potential influence of estrogen levels on cervical epithelial malignant transformation.

To reduce model complexity, we prioritized candidate genes with relatively stable performance and feasible IHC assessment for further model development. These genes have reported oncological relevance, which provides biological support for their inclusion in the model. The three-gene model achieved an AUC of 0.831 in the public progression dataset, suggesting favorable discriminatory performance in that dataset. Compared to traditional biomarkers such as Ki-67 or p16INK4a, our model may provide additional risk-stratification information for CIN progression.

Previous studies have explored the use of clinical information to predict CIN progression. A prediction study on the risk of CIN2+ in a cervical screening population incorporated factors such as age, race, smoking status, type of medical insurance, marital status, income, and previous HPV test results, achieving an AUC of 0.81 (38). Other cervical cancer prediction models have incorporated clinical characteristics, HPV-related variables, cytology, or biomarkers such as p16/Ki-67 to support individualized risk stratification (39). These studies provide useful context for the development of risk-based prediction models in cervical cancer prevention, while also highlighting the importance of external validation, calibration assessment, and evaluation of clinical utility before routine implementation. In our study, we integrated transcriptomic screening-derived molecular markers with clinical information, allowing the model to provide a more comprehensive framework for exploring lesion progression risk. However, as highlighted in previous studies, the translation of omics-based findings into clinically applicable biomarkers remains challenging and requires careful attention to reproducibility and external validation (40). Additionally, all predictive factors included in our study were derived from routinely collected pathological samples and clinical data during standard cervical screening. This design increases the model’s clinical feasibility, although further external validation and prospective evaluation are required before real-world implementation.

This study also ranked risk factors based on the Boruta algorithm and utilized Cox regression to construct prognostic curves, assessing the association of each predictor with disease progression. First, the protein products corresponding to the three core genes, which demonstrated significant predictive potential in public datasets, also exhibited predictive performance in our real-world cohort. This consistency between gene expression and protein level changes highlights their potential value as candidate markers for CIN progression prediction. Second, colposcopy examination revealed that the extent of cervical quadrant involvement showed an apparent inverse association with disease progression. This finding should be interpreted cautiously, as it is inconsistent with the conventional understanding that more extensive cervical involvement may reflect greater disease burden. Prior studies have suggested that a higher number of involved quadrants correlates with greater CIN severity due to a larger affected cervical surface area. Previous studies have also indicated that cervical lesion size and topographical distribution are clinically relevant and may affect cytological severity, CIN grade, and the diagnostic accuracy of colposcopy-directed biopsy (41, 42). However, it is important to note that within the same CIN grade, the relationship between lesion size and progression risk remains unconfirmed. Therefore, this finding should be interpreted cautiously rather than as evidence of a true protective biological effect. It may reflect residual confounding, selection bias, differential clinical management, or statistical instability related to the relatively small sample size and limited number of progression events. Further validation in larger cohorts is required. The impact of bacterial vaginosis (BV) on CIN progression has been widely reported. Studies suggest that BV-induced chronic inflammation may lead to DNA damage in cervical epithelial cells, increasing their susceptibility to genetic mutations and malignant transformation (43). Additionally, BV may contribute to an inflammatory microenvironment conducive to lesion progression by secreting pro-inflammatory cytokines such as IL-1β, TNF-α, and IL-6 (44). These findings are consistent with our observation and underscore the need to consider BV’s role in CIN prevention and management. Lastly, multiple HPV infections were identified as a potential risk factor in our feature importance ranking but were not included in the final model. Previous studies suggest that patients with multiple HR-HPV infections, especially those involving HPV16, HPV18, and other HR subtypes, have an increased risk of cervical cancer (45). This may be due to the activation of immune evasion mechanisms, whereby HPV co-infections alter the local cervical immune microenvironment, reducing anti-HPV immune responses and promoting persistent infection (46). However, conflicting evidence also exists (47, 48). Potential confounders such as HPV genotype variations, immune responses, and comorbidities may influence CIN progression but were not fully addressed due to limited data. In addition, comprehensive HPV-related variables, such as genotype-specific persistence and viral load, were not consistently available and were not incorporated into the final model, which may have limited the completeness of risk prediction.

The clinical cohort in this study was relatively small, with a limited number of progression events, which may have increased the risk of overfitting and contributed to instability in some effect estimates. Boruta-based feature selection identified five predictors for Cox model construction. With 49 progression events, the approximate events-per-variable ratio was 9.8, close to the conventional threshold of 10. However, events-per-variable thresholds are empirical and should not be interpreted as eliminating the risk of overfitting (49–52). In addition, no penalized regression or formal coefficient shrinkage method was applied in the primary analysis. Although Boruta-based feature selection was used to reduce the candidate predictor set, Boruta is not equivalent to coefficient shrinkage and does not replace penalized regression methods such as LASSO, ridge regression, or elastic net. Some relatively large hazard ratio estimates should therefore be interpreted cautiously, and coefficient instability and overfitting cannot be fully excluded. In particular, the relatively large hazard ratio estimate observed for Eag1 should be interpreted with caution, as its magnitude may partly reflect statistical instability related to the limited sample size and number of progression events. Future studies should evaluate penalized Cox regression or shrinkage approaches in larger independent cohorts.

Moreover, external validation with independent cohorts was not conducted. Therefore, the current model should be regarded as a preliminary internally validated prediction model, and its reproducibility, calibration, and clinical utility in diverse populations and real-world clinical settings remain to be established. Calibration was not formally assessed using calibration plots, calibration slope/intercept, or Brier score. Therefore, although the model showed potential discriminatory performance, the agreement between predicted and observed progression risks remains uncertain. Because well-calibrated risk estimates are essential for clinical decision-making, particularly when predicted probabilities are used to guide follow-up intensity or intervention thresholds, comprehensive calibration assessment is needed in future validation studies (53, 54). Decision curve analysis suggested potential clinical net benefit across selected threshold probabilities; however, these findings reflect model-based estimates and should not be interpreted as proof of real-world clinical benefit. Although the nomogram showed promising predictive value, several challenges may affect its implementation in routine colposcopy clinics. The molecular markers require standardized immunohistochemical assessment, reproducible scoring criteria, quality control, and acceptable turnaround times before clinical application. In addition, integration of the model into busy clinical workflows may require digital decision-support tools, such as a web-based calculator or electronic medical record integration. Future multicenter external validation and prospective evaluation are therefore needed before routine clinical implementation.

## 5 Conclusions

This study developed a novel risk assessment model by combining CIN immunohistochemical markers with clinical data, offering promising predictive value and potential clinical relevance. This model may help support risk stratification of LSIL patients and identify individuals who may require closer follow-up. However, the single-center design and limited sample size may limit generalizability. Larger multicenter studies are needed to confirm the model’s predictive performance, generalizability, and clinical utility.

## Data Availability

The publicly available datasets analyzed in this study are available in the NCBI Gene Expression Omnibus (GEO; https://www.ncbi.nlm.nih.gov/geo/) under accession numbers GSE27678, GSE63514, and GSE75132.
